# Physicians’ perspectives on cancer-related fatigue management and their suggestions for improvements in medical training: a cross-sectional survey study in Germany

**DOI:** 10.1007/s00520-024-08978-2

**Published:** 2024-11-14

**Authors:** Anna S. Wagner, L. Wehlen, Marlena Milzer, Martina E. Schmidt, Senta Kiermeier, Imad Maatouk, Karen Steindorf

**Affiliations:** 1 Department of Internal Medicine II, Section of Psychosomatics, Psychotherapy and Psychooncology, University Hospital Würzburg, Würzburg, Germany; 2 Department of Psychosomatic Medicine and Psychotherapy, Central Institute of Mental Health, Medical Faculty Mannheim/Heidelberg University, Mannheim, Germany; 3 University Hospital Mannheim, Mannheim Cancer Center, Heidelberg University, Mannheim, Germany; 4 Division of Physical Activity, Prevention and Cancer (C110), German Cancer Research Center (DKFZ), Heidelberg, Germany; 5 Division of Physical Activity, Prevention and Cancer, National Center for Tumor Diseases (NCT), NCT Heidelberg, a partnership between DKFZ and University Medical Center Heidelberg, Heidelberg, Germany

**Keywords:** Cancer-related fatigue, Cross-sectional design, Physicians, Supportive care, Survey study

## Abstract

**Purpose:**

Contrary to guidelines, many cancer patients are not screened for cancer-related fatigue (CRF) and do not receive information or adequate treatment. As physicians play a key role in cancer therapy, their knowledge of this common sequela and perspectives on its management are of major interest.

**Methods:**

For an online survey, physicians working in oncology in Germany were systematically drawn from registers and invited by using institutional newsletters or colleagues. Descriptive analyses, logistic regression analysis of physicians’ knowledge, and Mann‒Whitney* U* tests were performed.

**Results:**

Two-thirds of the 148 surveyed physicians felt (rather) well informed about CRF and capable of counseling patients. Only 32% of the sample were aware of CRF-specific guidelines. Despite of this, participants rated the scientific evidence for recommending physical activity, exercise programs, and psychotherapeutic interventions in accordance with guidelines as being mostly (very) strong. However, despite 82.4% of the physicians being (rather) aware of its evidence, only 56.1% often to almost always recommended psychotherapeutic interventions. CRF was rarely covered in medical studies and medical specialist training. The completion of advanced training for palliative care increased the likelihood of knowing guidelines (OR = 2.6, 95% CI [1.1–6.0], *p* < 0.05). Suggestions for improving training included the mandatory coverage of CRF in medical training or its consideration in interprofessional supportive care workshops.

**Conclusion:**

Although awareness and recommendation rates were adequate for some interventions in CRF treatment (such as physical activity), there were lower recommendation rates for others, including psychotherapy. Studies are required assessing for the reasons of this knowledge-to-practice gap. Moreover, training is needed among physicians in order to enhance knowledge of CRF guidelines.

**Trial registration:**

Clinicaltrials.gov, identifier: NCT04921644. Registered in June 2021.

## Introduction

Due to improvements in the early detection and treatment of cancer, the number of cancer survivors has significantly increased in recent years [1]. However, related side effects can impair the quality of life and daily functioning of cancer survivors beyond the completion of therapy [2–4]. One of the most distressing side effects is cancer-related fatigue (CRF) [5, 6], which is a symptom cluster that is described by the National Comprehensive Cancer Network (NCCN) as “a distressing, persistent, subjective sense of physical, emotional and/or cognitive tiredness or exhaustion related to cancer or cancer treatment that is not proportional to recent physical activity and that interferes with usual functioning” [7]. Up to one-third of patients struggle with CRF years after therapy completion, although this is a smaller proportion compared to patients with CRF during active cancer treatment [8, 9]. The assumed multiple causes of its development [10] as well as the lack of visibility and operational capability of symptoms seem to complicate adequate diagnostics and treatment [11–13].

CRF still appears to be undertreated, although evidence-based clinical practice guidelines have been published to guide healthcare professionals (HCPs), such as those from the Canadian Association of Psychosocial Oncology (CAPO) [14], the NCCN [7], or the European Society for Medical Oncology (ESMO) [15]. According to these guidelines, cancer patients should receive information about CRF, regular screening, and comprehensive diagnostics. Regarding CRF treatment, guidelines favor nonpharmacological interventions, particularly exercise programs, physical activity, and psychological interventions [16], rather than pharmacological treatment options, which are not commonly recommended [8].

However, in a recent study with 2,508 German cancer survivors, more than half reported of not feeling well informed about CRF. Forty percent stated that they had never been asked about fatigue by their treating physician [17]. Our own survey among 1,179 patients (five months after cancer diagnosis) demonstrated even more worrying results; specifically, almost 55% reported that their doctors had not asked them about whether and how much they felt exhausted [18]. Systematic screening with validated instruments and education, as suggested by the guidelines, were received by only 10–20% of the patients [18].

In international studies, a lack of knowledge has been discussed as being one contributing factor explaining these shortcomings [13, 19]. HCPs were unaware of CRF-related guidelines or not aware of content and struggled with recommending interventions, especially if outside of their specialty [13]. Overall, they lacked confidence in assessing and managing CRF [19]. As long as these gaps persist, the implementation gap is assumed to remain [13].

To date, there have been no studies examining the knowledge and perspectives of physicians in terms of CRF in Germany, though they are the main operators in cancer care. To enhance the overall viewpoint of this scenario the present study focused on physicians’ knowledge of CRF and corresponding guidelines, self-efficacy in counseling, coverage of CRF in medical education and training, and suggestions on how to improve education on CRF.

## Methods

### Study design

As part of the large-scale LIFT project (Longitudinal Investigation of Cancer-related Fatigue and its Treatment; Clinicaltrials.gov, identifier: NCT04921644), a cross-sectional online survey was conducted among physicians working in oncology in Germany. The data were collected from November 2021 to September 2022. For comparison reasons, this online survey was similar to two surveys conducted among psycho-oncologists [20] and nurses [21] within the LIFT project. Respective results regarding CRF knowledge among psycho-oncologists [20] and nurses [21] have been published elsewhere.

### Participants

This study aimed to recruit 210 physicians, including physicians working in oncology-related outpatient practices, inpatient oncology units, and oncological rehabilitation facilities (*n* = 70 for each institution) and seeing at least one cancer patient per week for at least one year.

Physicians working in oncology-related outpatient practices (i.e., board certified specialists for general medicine, hematology/oncology, gynaecology, gastroenterology, urology, pneumology) were identified from the registers of the Association of Statutory Health Insurance Physicians (Kassenärztliche Vereinigung, KBV) following random sampling. To encompass different regions in Germany, physicians working in outpatient practices were identified from seven federal states representing different oncology-associated disciplines. They were sent a postal invitation containing the online access link and were reminded by e-mail a few weeks later. Three selection waves were performed between November 2021 and July 2022, thus resulting in *n* = 210 contacted physicians from outpatient practices (participation rate < 10%). Subsequently, snowball sampling was used in August 2022 to request distribution at professional societies.

To recruit physicians working in inpatient oncology units, hospitals were systematically identified from the German Hospital Directory (Deutsches Krankenhaus Verzeichnis) between March and July 2022, resulting in 143 hospitals from the first and 114 from the second identification. Inpatient oncology units were contacted via phone or e-mail and asked to distribute the survey link among their staff. Reminder e-mails were sent several weeks later.

To reach the anticipated sample size of physicians working in rehabilitation, a stepwise recruitment procedure was applied between November 2021 and July 2022 considering the website of rehakliniken.de (7 facilities), the mentioned Hospital Directory (55), and the registry of the German Pension Insurance (Deutsche Rentenversicherung, DRV) (12). Facilities were contacted via e-mail or phone with the request to distribute the survey to their staff and were reminded via phone several weeks later. In August 2022, the survey was additionally distributed via the mailing lists of professional societies, as well as to personal contacts in rehabilitation facilities.

### Data collection

In accordance with a questionnaire from an Australian study [22] and under consideration of the literature [7, 23], we developed a self-report questionnaire. After having pretested the questionnaire two physicians working in oncology confirmed clarity and comprehensibility of the items. Survey questions other than questions on sociodemographic and professional characteristics are outlined below. Where necessary, the options ‘unable to judge’ and/or ‘other’ were provided for user-provided answers. The survey was conducted online via LimeSurvey® (LimeSurvey GmbH, Hamburg, Germany). Survey completion took 15 min, and the participants were remunerated with a total of €15.

Initiated by the question “How well do you currently feel informed about CRF?”, the perceived CRF-related knowledge was assessed with the answer options ‘very poorly’, ‘rather poorly’, ‘rather well’, and ‘very well’. Physicians further evaluated the statement “I think the majority of physicians are well informed about CRF.” on a four-point Likert scale. Their perceived self-efficacy in counseling on CRF was estimated on the basis of their agreement with the statement “I think that I can competently inform and counsel on CRF in my daily work” on a four-point Likert scale.

Physicians’ knowledge of CRF interventions was described based on their rating of the empirical level of evidence of listed interventions on a five-point Likert scale, ranging from ‘very strong evidence against’ to ‘very strong evidence for’ recommending an intervention. The evidence rating was followed by the question of how often they recommend those interventions to patients presenting with CRF symptoms on a five-point Likert scale, ranging from ‘never’ to ‘almost always’. Physicians’ knowledge of CRF-related guidelines was determined by using the binary question “Do you know (national or international) guidelines for CRF?”. This was specified by the question of how well they knew commonly used guidelines on a four-point Likert scale from ‘is not known to me’ to ‘contents and recommendations are well known to me’. If participants affirmed that they knew of any guidelines, their agreement with statements regarding the use of guidelines in clinical practice, such as ‘I don’t have enough time in my everyday work to read the comprehensive guidelines’, had to be rated on four-point Likert scales.

Finally, the coverage of CRF in medical education and training had to be assessed by using a three-point Likert scale (‘not at all/hardly’, ‘moderately’, and ‘comprehensively’), and participants’ suggestions were collected on how training could be improved with regard to CRF.

### Data analysis

Descriptive analyses were conducted for sample characteristics and CRF-related questions. Spearman’s correlations were calculated between knowledge of (CRF) guidelines and sociodemographic/ professional variables to indicate associations. Variables that were considered as potential determinants in univariate analyses and following theoretical reflection were simultaneously included in a binomial logistic regression to explore the associations between sociodemographic and professional variables and the likelihood of knowing CRF guidelines (yes/no). In addition, Mann‒Whitney *U* tests were performed to examine differences in the level of perceived knowledge and self-efficacy between physicians who knew CRF guidelines and those who did not. With respect to the Mann‒Whitney *U* tests, we calculated Pearson’s *r*, with |*r*|> 0.1 indicating small effects, |*r*|> 0.3 indicating moderate effects, and |*r*|> 0.5 indicating large effects. We used SPSS 29.0.0.0. for the statistical analyses, with *p* ≤ 0.05 (two-tailed) considered to indicate statistical significance.

## Results

### Sample

A total of 148 participants (70.8% of those who had provided informed consent in order to participate) completed the survey questionnaire. Complete questionnaires were included in the analyses. One-third of the participants were directly contacted by the study team, one-third through newsletters/mailing lists and one-third through colleagues. Seventy-five percent were working in acute care, 60.1% in palliative care, 50.7% in aftercare, and 15.5% in rehabilitation, with multiple answers being possible. Sociodemographic and professional characteristics of the sample are listed in Table 1.

**Table 1:** Demographic and professional data of the sample

	Total (*N* = 148)	
Characteristics	*M* or* n*	*SD* or* %*
Age (years)	44.3	12.1
< 40	67	45.3%
40–49	29	19.6%
50–59	29	19.6%
≥ 60	23	15.5%
Gender		
Male	83	56.1%
Female	65	43.9%
Work experience in oncology (years)	13.5	9.8
< 10	64	43.2%
10–20	45	30.4%
> 20	39	26.4%
Leadership position		
Yes	73	49.3%
No	75	50.7%
Workplace		
Certified cancer center^a^	88	59.5%
Oncological rehabilitation facility^b^	23	15.5%
Outpatient oncology practice^c^	12	8.1%
Other^d^	25	16.9%
Cancer patients per week		
1–10	25	16.9%
11–20	31	20.9%
21–30	38	25.7%
> 30	54	36.5%
Medical specialist training^e^ (completed)		
Oncology/Hematology	53	35.8%
Other	52	35.1%
Advanced training^f^ (completed)		
Psycho-oncology	8	5.4%
Palliative Care	42	28.4%
Other	24	16.2%

*M* mean, *n* number, *SD* standard deviation

^a^i.e., hospitals with inpatient oncology units which are specified in at least one cancer/organ entity and do fulfil the guidelines-based quality criteria of the German Cancer Society (DKG, Deutsche Krebsgesellschaft); 62.5% with completed medical specialist training (*n* = 55), 33.0% with at least one completed advanced training (*n* = 29)

^b^91.3% with completed medical specialist training (*n* = 21), 69.6% with at least one completed advanced training (*n* = 16)

^c^100% with completed medical specialist training (for oncology), 50% with at least one completed advanced training (*n* = 6)

^d^inpatient oncology units (*n* = 8), other inpatient units (*n* = 5), other outpatient practices (*n* = 10), other (*n* = 2)

^e^i.e., five years of training in a certain medical speciality (e.g., surgery, gynaecology, etc.)

^f^i.e., optional training to gain specific knowledge and skills within a medical speciality (e.g., regarding psycho-oncology or palliative care within the speciality of oncology)

### Perceived knowledge and self-efficacy to manage CRF

The majority of the physicians reported of feeling rather well informed (54.1%) or very well informed (9.5%). Accordingly, 66.9% (rather) agreed that they were capable of providing complete information and counseling for CRF. However, one-third still reported of feeling rather poorly informed (33.8%) or very poorly informed (2.7%).

### Knowledge on CRF treatment

Physicians’ knowledge of the scientific evidence of interventions used in CRF treatment is presented in Table 2. Almost all of the participants were aware of the scientific evidence of physical activity, with 79.7% rating the evidence levels for recommending it to CRF patients as very strong and 18.2% as strong. Slightly fewer participants estimated the evidence levels for recommending exercise programs in accordance with guidelines to be very strong (61.5%) or strong (30.4%). The majority of physicians were also convinced by (very) strong evidence levels for recommending yoga, mindfulness-based interventions, relaxation, and psychotherapeutic interventions. Regarding nutrition-based interventions, estimations varied, with more than half (52%) of them reporting the evidence for recommending it to be (very) strong, whereas 35.8% were unsure about it. Similarly, there was a more diverse range of opinions regarding the use of medication and mistletoe therapy in CRF treatment, with a tendency toward (very) strong evidence against recommending them.

**Table 2:** Physicians’ knowledge about the empirical evidence of interventions for CRF (*N* = 148)

	Very strong evidence against recommending	Strong evidence against recommending	Unclear evidence	Strong evidence for recommending	Very strong evidence for recommending	Unable to judge
Intervention	*n* (*%*)	*n* (*%*)	*n* (*%*)	*n* (*%*)	*n* (*%*)	*n* (*%*)
Physical activity in everyday life (e.g., taking a walk)	0 (0)	1 (0.7)	0 (0)	27 (18.2)	118 (79.7)	2 (1.4)
Exercise programs (e.g., strengths training, endurance training)	0 (0)	2 (1.4)	5 (3.4)	45 (30.4)	91 (61.5)	5 (3.4)
Yoga	0 (0)	0 (0)	23 (15.5)	68 (45.9)	46 (31.1)	11 (7.4)
Mindfulness-based interventions (e.g., qigong, MBSR)	0 (0)	2 (1.4)	31 (20.9)	56 (37.8)	41 (27.7)	18 (12.2)
Relaxation (e.g., PMR)	0 (0)	1 (0.7)	28 (18.9)	62 (41.9)	42 (28.4)	15 (10.1)
Medication	12 (8.1)	33 (22.3)	69 (46.6)	22 (14.9)	1 (0.7)	11 (7.4)
Psychotherapeutic interventions (e.g., behavioral therapy, psychoeducation)	0 (0)	3 (2.0)	13 (8.8)	66 (44.6)	56 (37.8)	10 (6.8)
Nutrition-based interventions (e.g., nutritional counseling)	1 (0.7)	6 (4.1)	53 (35.8)	52 (35.1)	25 (16.9)	11 (7.4)
Mistletoe therapy	22 (14.9)	32 (21.6)	52 (35.1)	12 (8.1)	5 (3.4)	25 (16.9)

*n* number of cases, *MBSR* mindfulness-based stress reduction, *PMR* progressive muscle relaxation

Almost all (96.7%) of the physicians (Table 3) indicated that they often or almost always recommend physical activity to patients presenting with CRF symptoms. Exercise programs were often or almost always recommended by 82.4% of the participants. More than half (56.1%) of the participants often or almost always advised their patients to try psychotherapeutic interventions; 37.2% to practice yoga. The recommendation rates for mindfulness-based interventions, relaxation and nutrition-based interventions varied. Medication was sometimes to almost always recommended by 27.7% of the physicians. Mistletoe therapy was less often recommended.

**Table 3:** Frequencies with which physicians recommended interventions in CRF treatment (*N* = 148)

	Never (0% of patients)	Rarely (1–25% of patients)	Sometimes (26–50% of patients)	Often (51–75% of patients)	Mostly/ almost always (76–100% of patients)	Unable to judge
Intervention	*n* (*%*)	*n* (*%*)	*n* (*%*)	*n* (*%*)	*n* (*%*)	*n* (*%*)
Physical activity in everyday life (e.g., taking a walk)	1 (0.7)	3 (2.0)	1 (0.7)	30 (20.3)	113 (76.4)	0 (0)
Exercise programs (e.g., strengths training, endurance training)	7 (4.7)	6 (4.1)	13 (8.8)	33 (22.3)	89 (60.1)	0 (0)
Yoga	22 (14.9)	28 (18.9)	42 (28.4)	22 (14.9)	33 (22.3)	1 (0.7)
Mindfulness-based interventions (e.g., qigong, MBSR)	36 (24.3)	35 (23.6)	29 (19.6)	21 (14.2)	22 (14.9)	5 (3.4)
Relaxation (e.g., PMR)	29 (19.6)	32 (21.6)	34 (23.0)	26 (17.6)	26 (17.6)	1 (0.7)
Medication	63 (42.6)	44 (29.7)	28 (18.9)	10 (6.8)	3 (2.0)	0 (0)
Psychotherapeutic interventions (e.g., behavioral therapy, psychoeducation)	13 (8.8)	16 (10.8)	36 (24.3)	47 (31.8)	36 (24.3)	0 (0)
Nutrition-based interventions (e.g., nutritional counseling)	26 (17.6)	35 (23.6)	34 (23.0)	36 (24.3)	16 (10.8)	1 (0.7)
Mistletoe therapy	114 (77.0)	17 (11.5)	5 (3.4)	7 (4.7)	1 (0.7)	4 (2.7)

*n* number of cases, *MBSR* mindfulness-based stress reduction, *PMR* progressive muscle relaxation

### Knowledge of CRF guidelines

In contrast to their knowledge of the scientific evidence levels of most CRF treatment options, more than three-quarters of the physicians reported of being unaware of any national or international CRF-related guidelines. When directly asked about specific guidelines, the majority indicated not knowing about the NCCN (60.8%), ESMO (54.7%), or CAPO (85.8%) guidelines. Regarding German guidelines, 68% reported of being unaware of the psycho-oncology guidelines themselves or their contents, whereas 31.8% acknowledged partial or good knowledge of them. The German palliative care guidelines, to which CRF is also referred, were partly or well known by 56.1% of the physicians.

Of the physicians who were aware of any guidelines, 62.9% agreed that guidelines recommendations are clear and detailed enough for use in clinical practice. Furthermore, half of the participants reported of noticeable gaps. Moreover, there was broad agreement on lack of time as a barrier to reading the CRF guidelines (67.0%), as well as on the requirement of compatibility with existing procedures when implementing guidelines (79.9%). On the question of whether training is necessary for effective implementation, participants were divided (39.8% disagreeing, 45.5% agreeing).

To determine which sociodemographic or professional variables most likely determine knowledge of (CRF) guidelines, a binomial logistic regression was performed. Preceding correlation analyses demonstrated a significant correlation between knowledge of (CRF) guidelines and the following variables: completed advanced training in palliative care (*r* = 0.276, *p* < 0.001), work experience in oncology (*r* = 0.214, *p* = 0.009), completed advanced training in psycho-oncology (*r* = 0.186, *p* = 0.023), and completed medical specialist training in oncology/hematology (*r* = 0.175, *p* = 0.033). After gender and age, these variables were included stepwise in the regression model. The analyses demonstrated that the completion of advanced training in palliative care was significantly associated with an increased likelihood of knowing CRF guidelines (OR = 2.6, 95% CI [1.1–6.0], *p* < 0.05). The remaining variables did not significantly contribute to the variance in knowledge of (CRF) guidelines.

The level of their perceived knowledge differed between physicians who knew about CRF guidelines (*M*_dn_ = 2.00) and those who did not (*M*_dn_ = 2.00) (*Z* =  − 4.161, *p* < 0.001, *r* =  − 0.342), with those who were aware of guidelines perceiving their knowledge of CRF as being higher. Similarly, the level of self-efficacy differed between the two groups (*M*_dn_ = 2.50 vs. *M*_dn_ = 2.00), with those who were aware of guidelines perceiving higher self-efficacy levels (*Z* =  − 5.464, *p* < 0.001, *r* =  − 0.451).

### CRF in medical education and training

Ninety-one percent of the participants stated that the topic of CRF was not at all or hardly covered in their medical studies. Nevertheless, 65.4% felt that this scenario applied to medical specialist training. However, according to one-third of the participants, advanced training, e.g., in psycho-oncology or palliative care, covered CRF at least moderately (58.8%) to comprehensively (14.7%). Physicians suggested including mandatory sessions in the medical studies and medical specialist training for oncology/hematology with a focus on the etiology of CRF, its diagnostic process, and treatment options. They further highlighted the sensitization to CRF, specifically regarding its high prevalence and early onset in cancer treatment and during hospitalization. Additionally, participants suggested the publication of CRF information in professional journals and respective internet portals, as well as in local circles or congresses. Emphasizing interdisciplinary cooperation, participants wished for more exchange of ideas, such as in the context of interprofessional workshops on supportive care. Finally, physicians called for information leaflets about services and treatment options available in the local area.

## Discussion

This study aimed to explore physicians’ knowledge and attitudes regarding CRF, its management, and corresponding clinical practice guidelines. Moreover, its coverage in medical education and training was studied with the aim of deriving suggestions for improvements.

The majority of the physicians felt rather informed and capable of competently counseling patients. Accordingly, in a previous study on the knowledge of HCPs of CRF, physicians had greater knowledge of CRF than other HCPs [23]. Contrary to our results, in a study among HCPs working in palliative care participants did not feel confident in assessing and managing CRF [19]. As only HCPs from inpatient palliative care were considered in that study, CRF management may be more challenging if patients are limited in mobility [24]. However, even if most of our participants felt rather informed about CRF, a considerable proportion still felt poorly informed.

Most physicians were unaware of any (inter)national CRF guidelines. Among international guidelines, those from the ESMO were best known to participants. National guidelines, which at least partially cover CRF, were more (if not sufficiently) familiar to physicians. Similarly, in a survey among HCPs in Australia CRF-related guidelines were used by less than a quarter in daily clinical practice [22]. Accordingly, the physicians in our study mentioned a lack of time to read the comprehensive guidelines, as well as noticeable gaps in clinical use. This observation is supported by Pearson, et al. [25], wherein HCPs also indicated a lack of practical details and clinical tools in the CAPO guidelines. Additionally, a majority of our participants acknowledged that guidelines implementation requires compatibility with existing procedures, which is also similar to the results of Pearson, et al. [25] However, almost 40% of our participants disagreed with the necessity of guidelines training for effective implementation. It is hypothesized that a lack of time in healthcare is one cause underlying this disagreement. This could further hint at low expectations regarding a single training session and the need for more practical and steady support, such as with the use of pocket guidelines or checklists. However, there are promising results from a brief one-time training on CRF guidelines among HCPs [26]. The importance is highlighted by the fact that the completion of advanced training for palliative care among our sample population increased the likelihood of knowing guidelines, which was correspondingly associated with a greater level of perceived knowledge and self-efficacy.

Despite their poor knowledge of (CRF) guidelines, physicians were quite aware of physical activity, exercise programs, and psychotherapy as being effective interventions to reduce CRF. For physical activity, this is not only discernible in the rating of its scientific evidence but also in a high recommendation rate, including a high recommendation frequency. Even if lower, the recommendation rate for exercise programs was also acceptable. However, as both physical activity and supervised exercise programs have highly supporting evidence, exercise programs should generally be offered as an option in addition to physical activity. Although the evidence ratings in the cohort of psycho-oncologists were similar to those of physicians for the three intervention groups, among psycho-oncologists an even smaller percentage recommended exercise programs to the majority of their patients, thus indicating a knowledge-to-practice gap [20].

Among physicians a knowledge-to-practice gap was observed regarding psychotherapeutic interventions. Although more than 80% of participants reported of knowing about the efficacy of psychotherapeutic interventions, only half of them recommended them, which is quite alarming. Similar findings among HCPs have also been reported in a previous study [12]. In conjunction with our results, Senf, et al. [27] reported of positive beliefs among oncologists about the efficacy of psycho-oncology regarding emotional distress in cancer patients, whereas psycho-oncological issues were covered in less than every second consultation. This leads to the question why, despite the knowledge and evidence, are physicians not recommending psychotherapy as frequently as physical activity or exercise programs? In Senf, et al. barriers were primarily perceived on the patient side; e.g., patients either refused to talk about emotional distress or refused psycho-oncological counseling [27]. Accordingly, only 28.9% of 4,020 cancer patients in Germany reported of the use of psychotherapy and/or psychological counseling in terms of cancer-related distress [28]. Another major barrier and explanation to the identified knowledge-to-practice gap in our study might be a lack of resources and/or staff to provide psychotherapeutic interventions to most patients with CRF. Further on, there might be practical challenges for some patients with advanced illness in participating in psychotherapy. One could also hypothesize that physicians might feel unsure about how to recommend psychotherapy or communicate with patients about it in terms of CRF as a sequela of cancer with often rather unclear underlying causes. Due to the assumed multiple causes of CRF and its tremendous impact on patients’ quality of life, barriers on HCPs side to recommend psychosocial interventions as well as to use these interventions on the patient side need to be considered. Psycho-social support should be suggested more often.

Finally, yoga and other mind–body interventions as further promising methods should be generally offered and discussed with patients presenting with CRF symptoms [29–31]. However, as previously reported in Martin, et al. [12], less than half of our participants recommended those interventions, and likewise seemed to be unsure about the corresponding scientific evidence. Apart from insufficient knowledge, certain (negative) representations of mind–body interventions may be causal for recommending them only to selected individuals, thus resulting in low recommendation rates.

### Clinical implications

The need for guidelines-orientated training on CRF becomes apparent. As guidelines are based on current research, physicians may rely more easily on the provided information due to increased confidence. Participants themselves called for more opportunities to participate in CRF workshops. Those should offer information on CRF in a broader theoretical context, e.g., being covered in physical and psychosocial long-term effects of cancer (treatment) and in an interprofessional setting. Comprehensive patient information about the etiology of CRF and local treatment options may further help HCPs in counseling as well as skills training on how to recommend interventions, e.g., psychotherapy, to patients with CRF. Especially for psychotherapeutic interventions, if resources are lacking, respective structures need to be built. Overall, physicians need to be encouraged to devote more attention to the management of CRF.

### Study limitations

This is the first study in Germany investigating CRF knowledge among physicians and their perspectives on current CRF management. Due to various approaches employed in recruitment, we could invite physicians working in different care settings throughout Germany to participate in the study. Nevertheless, the abandonment of random sampling at one point during the recruitment process (due to the fact that physicians were too hard to contact) can be seen as being limiting to our study results. Moreover, a selection bias cannot be ruled out. It is reasonable to hypothesize that participants in this study were more interested in the topic and thereby more knowledgeable than the average physician in Germany. Consequently, the lacking knowledge of (CRF) guidelines, as well as the identified knowledge-to-practice gap, may be more pronounced outside of this sample. The small size of some subgroups, such as in workplaces, further prevented more comprehensive analyses. However, due to the significance of rehabilitation facilities in CRF management, it may be of interest to explore more precisely the CRF-related knowledge of physicians in rehabilitation.

## Conclusion

The majority of physicians were unaware of any CRF-related clinical practice guidelines. The likelihood of knowing guidelines was greater if participants had been trained in palliative care. The coverage of CRF in medical studies and medical specialist training was lacking. Nonetheless, physicians were generally aware of the scientific evidence that exercise programs and physical activity are effective interventions for reducing CRF. This was also reflected in the high recommendation rates, with physical activity being slightly more often recommended than exercise programs. Awareness of the effectiveness of psychotherapeutic interventions did not correspond to the recommendations, thus indicating a clear knowledge-to-practice gap. Further studies are required to assess for the reasons of this gap.

## Data Availability

The data that support the findings of this study are available from the corresponding author, K.S., upon reasonable request.
